# Morphological, densitometric and mechanical properties of mandible in 5-month-old Polish Merino sheep

**DOI:** 10.1186/s12917-016-0921-3

**Published:** 2017-01-05

**Authors:** Anna Szabelska, Marcin R. Tatara, Witold Krupski

**Affiliations:** 1 Department of Prosthetic Dentistry, Medical University in Lublin, ul. Karmelicka 7, 20-081 Lublin, Poland; 2 Department of Animal Physiology, Faculty of Veterinary Medicine, University of Life Sciences in Lublin, ul. Akademicka 12, 20-950 Lublin, Poland; 3 II Department of Radiology, Medical University in Lublin, ul. Staszica 16, 20-081 Lublin, Poland

**Keywords:** Sheep, Mandible, Volumetric bone mineral density, Quantitative computed tomography (QCT), Animal model

## Abstract

**Background:**

Among bones building the axial and appendicular skeleton, the mandible is characterized by unique morphological and functional traits. The aim of the study was to evaluate morphological, densitometric and mechanical properties of mandible in 5-month-old Polish Merino sheep. Using quantitative computed tomography, volumetric bone mineral density (vBMD) and calcium hydroxyapatite density of the cortical bone (Cb_Ca-HA_), mean vBMD (MvBMD) and total bone volume were determined. Using computed tomography cross-sectional scans of the mandible, cross-sectional area, second moment of inertia, mean relative wall thickness and cortical index were determined. Three-point bending test was applied to determine mechanical properties. Serum concentration of insulin-like growth factor I (IGF-I) and bone-specific alkaline phosphatase (BAP) was also measured.

**Results:**

All the investigated morphometric, densitometric and mechanical parameters of the right and left mandibular halves were not significantly different (*P* > 0.05). There was no correlation of final body weight, MvBMD, Cb_Ca-HA_, BAP and IGF-I with all the analyzed parameters of mandible (*P* > 0.05). However, positive correlations between the other investigated morphometric, densitometric and mechanical parameter of mandible were found (*P* < 0.05).

**Conclusions:**

Relationships between morphological, densitometric and mechanical parameters of the mandible indicate that the elaborated experimental model may serve for further studies on metabolic responses of skeletal system to physiological, nutritional, pharmacological, toxicological and environmental factors.

## Background

Osteoporosis in animals and humans is a metabolic disorder of skeletal system characterized by low bone mass and microarchitectural deterioration of bony tissue with consequent increased risk of bone fracture [1–3]. Considering that peak bone mass (PBM) in mature mammals is genetically determined in 70–80%, approximately 20–30% of the variation in PBM is determined by environmental factors such as nutrition, physical activity, exposure to sunlight, external temperature and other [4]. Thus, manipulations of the environmental factors during growth and development period in vertebrates may result in the attainment of higher bone mass of skeletal system expressed by superior bone mineral density, morphological and the mechanical properties of bones [5]. Among bones building the axial and appendicular skeleton, mandible is characterized by unique morphological and functional traits. Whereas vertebral bodies consist mainly trabecular bone, long bones are composed of trabecular and cortical bone types [6]. Mandible in mammals is built of cortical bone mainly and it is equipped with teeth physiologically [7]. It is susceptible to bone loss occurring with age and in response to endocrine and metabolic impairments, medications or locally acting factors [8–11]. In humans, after 50 years of age, there is an observed bone loss of the cortical bone compartment with subsequent thinning of the lower jaw and an increase in its porosity. The rate of mandibular bone loss is sex-dependent, with females having a lower mandibular size, volume and bone mass compared to males [12, 13]. Considering the importance of mandibular functions in animals and humans, the aim of the study was to elaborate original experimental model for experimental studies focused on bone metabolism regulation. There is a dearth of information on the basic anatomical and physiological characteristics of the mandible of lambs. Thus, in this study, morphological, densitometric, and mechanical properties of mandible in 5-month-old Polish Merino male sheep was investigated.

## Methods

### Experimental design and sampling procedure

The study was carried out on growing Polish Merino male lambs (*N* = 7) which were reared intensively from birth to 5 months of age under standard rearing conditions. Lambs and ewes were provided with drinking water *ad libitum* and fed a standard diet. During neonatal and postnatal period (up to 10 weeks of life) ram lambs were fed maternal milk. Between the fourth week and the fifth month of life, lambs were fed commercial concentrate and hay *ad libitum*. At the age of 5 months of life, the animals were euthanized to obtain whole mandible for morphometric, densitometric and mechanical analyses. Bone samples were isolated postmortally and cleaned of remaining soft tissues, including periosteum. Morphological properties of the whole mandible (including teeth) such as bone weight and bone length were measured. To avoid sample dehydration, mandibles were wrapped in plastic bags and stored frozen at –25 °C until further analyses. Bone samples were thawed at room temperature for 2 h before further morphometric, densitometric and mechanical analyses.

### Densitometric evaluation of mandible

**Fig. 1:**
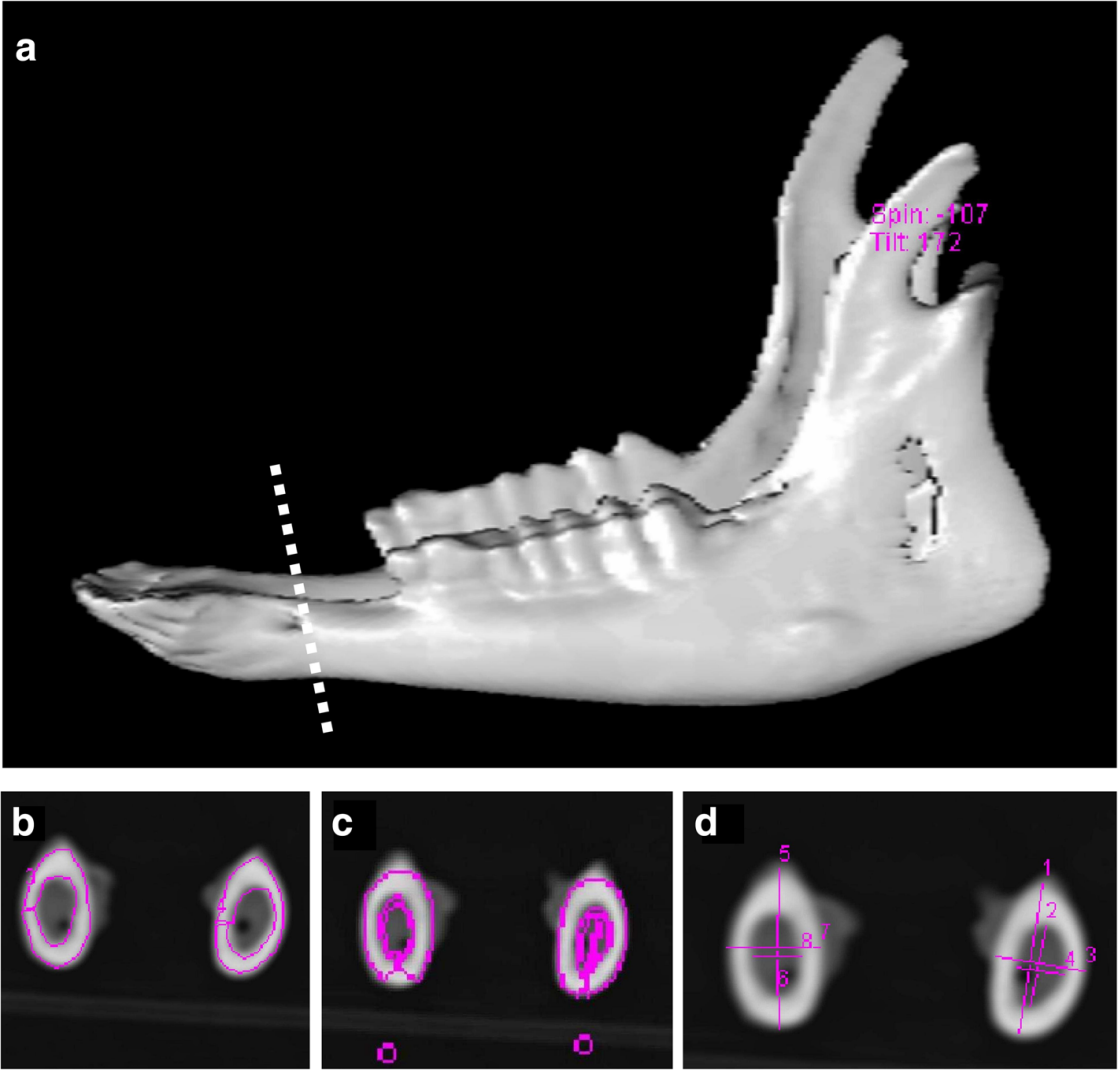
Three dimensional (3D) computed tomography reconstruction of mandible in 5-month-old Polish Merino sheep showing the placement of the measuring scans (**a**). Measurement of volumetric bone mineral density (vBMD) of the cortical bone (Cd) of the right and left mandibular body on cross-sectional measuring scans (**b**) using quantitative computed tomography (QCT). Volumetric bone mineral density of the cortical bone of mandible was measured on 2 mm thick scans positioned in the half distance of the diastema (half distance between canine (the fourth incisor) and premolar). Calcium hydroxyapatite density of the cortical bone (Cb_Ca-HA_) was measured at the same position on 10 mm thick cross-sectional measuring scan of mandible (**c**). Results of Cb_Ca-HA_ measurements were expressed in mg of Ca-HA/ml. Geometrical parameters of the right and left mandibular body were determined on the basis of measurements of horizontal and vertical diameters (both external and internal) of the mid-diastemal cross-sections of the bone obtained from computed tomography multiplanar reconstructions (**d**). All these measurements were performed using Somatom Emotion Siemens scanner supplied with Somaris/5 VB10B software and Osteo CT application package (Siemens, Erlangen, Germany)

Quantitative computed tomography (QCT) technique and Somatom Emotion Siemens apparatus (Siemens, Erlangen, Germany), equipped with Somaris/5 VB10B software (version B10/2004A) were used to determine volumetric bone mineral density (vBMD) of the cortical bone (Cd), mean volumetric bone mineral density (MvBMD) and total bone volume (Bvol) of the whole mandible with teeth (Volume Evaluation application package, Siemens, Erlangen, Germany). The measurement of Cd was performed on cross-sectional scan of mandibular body positioned in the half distance of the diastema (half distance between canine (the fourth incisor) and premolar, approximately 50 mm measuring from the extremity of the mandible determined by the external alveolar margin of the first lower incisor) (Fig. 1a and b). Using the same measuring scan and Osteo CT application package (Software Version B10/2004A), calcium hydroxyapatite (Ca-HA) density of the cortical bone (Cb_Ca-HA_) compartment was determined (Fig. 1c). Volumetric bone mineral density of the cortical bone of mandible and Cb_Ca-HA_ were measured on the same cross-sectional scans for both the right and left mandibular halves. Mean volumetric bone mineral density of mandible and Bvol were determined using volume-of-interest (VOI) limited by minimum and maximum density of the investigated samples at 0 and 3071 Hounsfield units, respectively. The measurements of MvBMD and Bvol were performed for the whole mandible (combined right and left halves) and the results obtained reflect MvBMD measured for all the anatomical structures including teeth.

### Determination of geometrical properties of mandible

Geometrical properties of the right and left mandibular body were determined on the basis of measurements of horizontal and vertical diameters (both external and internal) of the mid-diastemal cross-sections of the bone (the same measuring scans which were used for densitometric evaluation) obtained from computed tomography multiplanar reconstructions (Fig. 1d). The values of cross-sectional area (A), second moment of inertia (Ix), mean relative wall thickness (MRWT) and cortical index (CI) were determined in accordance to the following mathematical formulas:

$$ \mathrm{A}=3.14\left(\mathrm{H}\mathrm{V}\hbox{--} \mathrm{h}\mathrm{v}\right)/4 $$

$$ \mathrm{I}\mathrm{x}=3.14\left({\mathrm{V}}^3\mathrm{H}\hbox{--} {\mathrm{v}}^3\mathrm{h}\right)/64 $$

$$ \mathrm{MRWT}=\left[\left(\mathrm{V}\hbox{--} \mathrm{v}\right)/\mathrm{v} + \left(\mathrm{H}\ \hbox{--}\ \mathrm{h}\right)/\mathrm{h}\right]/2 $$

$$ \mathrm{C}\mathrm{I} = \left\{\left[\mathrm{H}\hbox{-} \mathrm{h}/\mathrm{H}+\left(\mathrm{V}\ \hbox{--}\ \mathrm{v}\right)/\mathrm{V}\right]/2\right\}100 $$

where: H – horizontal external diameter, h – horizontal internal diameter, V – vertical external diameter, v – vertical internal diameter [14–16].

### Mechanical analysis of mandible

Using an INSTRON 3367 apparatus and Bluehill 2 software (Instron Corp., Canton, USA) the relationship between the loading force and bone displacement in the three-point bending test was recorded. Right and left halves of mandible (separated at the mandibular symphysis) were placed separately on flat bone supports on the lateral mandibular surface and the measuring head loaded bone samples on the medial surface at 50% of its length with the constant speed of 50 mm/min. Mechanical parameters such as maximum elastic strength (Wy) and ultimate strength (Wf) of both mandibular halves were determined. The distance between bone supports was set at 40% of mandible length.

### Biochemical analysis of serum

Blood samples were collected from the lambs at 5 months of age for serum analysis. Insulin-like growth factor I (IGF-I) was determined in serum using IGF-I ELISA kit and two-site IEMA (Immunodiagnostic Systems Ltd., Boldon, Tyne & Wear, UK). Serum concentration of bone-specific alkaline phosphatase (BAP) was measured using Ostase® BAP immunoenzymometric assay (IEMA; Immunodiagnostic Systems Ltd., Boldon, Tyne & Wear, UK). Results of biochemical evaluation of serum were obtained with the use of Benchmark Plus microplate spectrophotometer supplied with Microplate Manager Software Version 5.2.1 (Bio-Rad Laboratories, Inc., Hercules, CA, USA).

### Statistical analysis

Statistical analysis of data collected was performed using Statistica software (version 6.0). Data are presented as means ± SEM. The comparison of mean values of the investigated variables between right and left halves of the mandible was performed using paired Student’s *t*-test for dependent variables. *P*-value < 0.05 was considered as statistically significant for all comparisons. Pearson's correlation coefficient (r) was determined between all the investigated mandibular variables, final body weight, IGF-1 and BAP serum concentrations and *P* < 0.05 was considered as statistically significant.

## Results

Results of morphological, densitometric and mechanical properties of the mandible in 5-month-old ram lambs are presented in Table 1. All the investigated morphometric, densitometric and mechanical parameters of right and left mandibular halves were not significantly different (*P* > 0.05). The values of Pearson’s correlation coefficient between all the investigated parameters of mandible, final body weight, and serum concentration of BAP and IGF-1 in 5-month old sheep are shown in Table 2. Mandibular weight was observed to be positively correlated with mandibular length, Bvol, Cd, A, Ix and Wy (*P* < 0.05). Mandibular length was positively correlated with Bvol and Cd (*P* < 0.05). Total bone volume was positively correlated with Cd, A, Ix, and Wf (*P* < 0.05). Cross-sectional area and Ix were found to be positively correlated (*r* = 0.97; *P* < 0.001) and both parameters were positively correlated with Cd (*P* < 0.05). Volumetric bone mineral density of the cortical bone was found to be also positively correlated with Wy and Wf (*P* < 0.05). Positive correlations between MRWT and CI as well as between Wy and Wf were found (all *P* < 0.05).

**Table 1:** Morphometric, densitometric and mechanical properties of mandible in 5-month-old Polish Merino sheep

Investigated parameter	Right mandible	Left mandible	Whole mandible
Bone weight (g)	-	-	116.3 ± 5.6
Bone length (mm)	218.9 ± 2.8	218.6 ± 2.9	218.7 ± 2.9^a^
Total bone volume (cm^3^)	-	-	71.7 ± 3.3
Volumetric bone mineral density of the cortical bone (g/cm^3^)	2.457 ± 0.027	2.446 ± 0.025	2.451 ± 0.024^a^
Calcium hydroxyapatite density of the cortical bone (g/ml)	1043 ± 26	1065 ± 35	1054 ± 26^a^
Mean volumetric bone mineral density (g/cm^3^)	-	-	1.864 ± 0.017
Cross-sectional area (mm^2^)	51.7 ± 1.9	56.0 ± 2.8	53.9 ± 2.3^a^
Second moment of inertia (mm^4^)	254 ± 21	276 ± 22	265 ± 21^a^
Mean relative wall thickness	0.756 ± 0.020	0.843 ± 0.025	0.797 ± 0.017^a^
Cortical index	42.9 ± 0.6	45.6 ± 0.7	44.3 ± 0.5^a^
Maximum elastic strength (N)	801 ± 31	844 ± 69	823 ± 42^a^
Ultimate strength (N)	1121 ± 105	1312 ± 135	1216 ± 106^a^

Values are means ± standard error of the mean

^a^Values obtained as means of right and left halves of mandible

**Table 2:** The values of Pearson’s correlation coefficient between the investigated parameters of mandible, final body weight and serum concentration of bone-specific alkaline phosphatase (BAP) and insulin-like growth factor 1 (IGF-1) in ram lambs at the age of 5 months

Investigated parameter	Body weight	Mandibular weight	Mandibular length	MvBMD	Bvol	Cd	Ca-HA	A	Ix	MRWT	CI	Wy	Wf	BAP	IGF-1
Body weight	x	0.58	0.45	- 0.06	0.51	0.72	0.32	0.75	0.68	0.39	0.38	0.65	0.55	- 0.61	- 0.64
Mandibular weight	0.58	x	0.77^a^	0.29	0.98^a^	0.91^a^	0.02	0.89^a^	0.94^a^	0.10	0.12	0.66	0.86^a^	- 0.42	- 0.50
Mandibular length	0.45	0.77^a^	x	0.69	0.81^a^	0.77^a^	0.35	0.61	0.62	0.08	0.10	0.59	0.68	- 0.47	- 0.07
MvBMD	- 0.06	0.29	0.69	x	0.33	0.40	0.69	- 0.01	- 0.02	0.22	0.24	0.51	0.50	0.15	0.46
Bvol	0.51	0.98^a^	0.81^a^	0.33	x	0.89^a^	- 0.01	0.88^a^	0.93^a^	0.03	0.04	0.61	0.80^a^	- 0.41	- 0.48
Cd	0.72	0.91^a^	0.77^a^	0.40	0.89^a^	x	0.37	0.88^a^	0.83^a^	0.43	0.44	0.80^a^	0.86^a^	- 0.27	- 0.44
Ca-HA	0.32	0.02	0.35	0.69	- 0.01	0.37	x	- 0.03	- 0.18	0.68	0.68	0.61	0.34	0.21	0.29
A	0.75	0.89^a^	0.61	- 0.01	0.88^a^	0.88^a^	- 0.03	x	0.97^a^	0.20	0.20	0.54	0.64	- 0.48	- 0.69
Ix	0.68	0.94^a^	0.62	- 0.02	0.93^a^	0.83^a^	- 0.18	0.97^a^	x	0.02	0.03	0.52	0.70	- 0.54	- 0.71
MRWT	0.39	0.10	0.08	0.22	0.03	0.43	0.68	0.20	0.02	x	0.99^a^	0.33	0.21	0.27	0.19
CI	0.38	0.12	0.10	0.24	0.04	0.44	0.68	0.20	0.03	0.99^a^	x	0.34	0.22	0.28	0.20
Wy	0.65	0.66	0.59	0.51	0.61	0.80^a^	0.61	0.54	0.52	0.33	0.34	x	0.90^a^	- 0.12	- 0.43
Wf	0.55	0.86^a^	0.68	0.50	0.80^a^	0.86^a^	0.34	0.64	0.70	0.21	0.22	0.90^a^	x	- 0.21	- 0.40
BAP	- 0.61	- 0.42	- 0.47	0.15	- 0.41	- 0.27	0.21	- 0.48	- 0.54	0.27	0.28	- 0.12	- 0.21	x	0.40
IGF-1	- 0.64	- 0.50	- 0.07	0.46	- 0.48	- 0.44	0.29	- 0.69	- 0.71	0.19	0.20	- 0.43	- 0.40	0.40	x

*MvBMD* mean volumetric bone mineral density, *Bvol* total bone volume, *Cd* volumetric bone mineral density of the cortical bone, *Ca-HA* calcium hydroxyapatite density of the cortical bone, *A* cross-sectional area, *Ix* second moment of inertia, *MRWT* mean relative wall thickness, *CI* cortical index, *Wy* maximum elastic strength, *Wf* ultimate strength

^a^Statistically significant values for *P* < 0.05

## Discussion

In this study, a new experimental model to investigate bone metabolism regulation and skeletal system properties in mammals was introduced. Using quantitative computed tomography technique, for the first time Bvol and MvBMD of mandible in sheep was determined. While Bvol represents morphometric parameter, MvBMD is a densitometric parameter characterizing all anatomical structures of the mandible, including the teeth. Similarly to bone weight determination, both Bvol and MvBMD measurements were performed for whole mandible without its separation into two halves. The methodological advantage resulting from the determination of Bvol and MvBMD for whole mandible is that both these parameters may be evaluated both ex vivo and in vivo, while mandibular weight may be determined precisely on ex vivo isolated samples only. As opposed to the parameters characterizing whole mandible, densitometric, geometric and mechanical properties in this bone were determined for the right and left halves separately. As the results of densitometric evaluation of the right and left mandibular body, it was shown that Cd and Ca-HA values were not significantly different in both these halves of the bone. Noteworthy is the fact that anatomical shape and structure of mandible in sheep and physiological occurrence of the diastema enable volumetric bone mineral density determination (in terms of Cd and Ca-HA) exclusively for cortical bone compartment. The reference measuring point of the densitometric parameters in mandible is recommended at the half length of the diastema, since at this point also geometrical parameters for the right and left halves may be evaluated [17]. In this study, computed tomography evaluations of serial cross-sections of the whole mandible along its longitudinal axis have not provided the reference point for the separate determination of vBMD of the trabecular bone compartment. Moreover, it was not possible to exclude non-invasively teeth from bone tissue of the mandible for measurements of bone weight, Bvol and MvBMD. As opposed to sheep model, studies in pigs have revealed that vBMD of the cortical bone in mandible may be measured on cross-sectional scan of the mandibular body positioned just after the 4^th^ premolar tooth [18]. It is interesting that at this reference point in mandible of pigs, cortical bone area and mechanical properties may be also evaluated. The loading head acts on the mandible in pigs exactly at this reference point in three-point bending test [10]. Mechanical testing of mandible in sheep using three-point bending test can not be performed in the reference point used for Cd and Ca-HA determinations, which makes this model different from the mandibular models in pigs. Both densitometric parameters are measured approximately 5 cm from proximal extremity of mandible in sheep (determined by the external alveolar margin of the first lower incisor), while the loading head should act on bone sample at 50% of its length [14, 16]. In this study, the length of mandible varied between 205 and 228 mm. The separation of the right and left halves of the mandible enabled their separate evaluation. Bone length measured for the right and left halve of mandible did not revealed significant differences. Analogical results were obtained evaluating geometrical parameters of mandible. Cross sectional area, Ix, MRWT and CI had no significant differences in the values obtained in both mandibular halves. As opposed to anatomical traits of mandible in other animal species like pig, dog, fox and rodents, oval shape of the cross section of the middle part of the diastema in sheep enables determination of geometrical parameters in accordance to the principles applied in case of long bones such as tibia, femur, humerus and ribs [16, 19–23]. While A and Ix are considered as geometrical indices of mechanical endurance of bone shaft to acting forces, MRWT and CI reflect the amount of bone tissue accumulated in the midshaft ring in relation to the medullary cavity size [14–16]. Besides morphological, densitometric and geometrical parameters, mechanical testing of both halves of mandible was performed in this study. Based on three-point bending test, neither Wy nor Wf were found to be differentiated between the right and left halves of mandible. Considering the observed lack of the differences of mechanical endurance of the right and left halves of mandible and the fact that mandible in sheep may be classified as flat bone, precise mechanical testing of the bone may include the three-point and four-point bending tests; however, in such case both halves undergo structural disintegration [24]. Separate evaluation of the right and left halves of mandible gives possibility to take average from both measurements, improving methodological precision. Moreover, separate evaluation of bone weight, Bvol and MvBMD for the right and left halves of mandible is possible; however, all these measurements must be repeated after the separation of bone halves at the symphysis.

In this study, the correlations between all the investigated parameters of mandible, final body weight and serum BAP and IGF-1 concentration were evaluated. Final body weight and serum indices of bone metabolism were not significantly correlated with the other investigated parameters of mandible in lambs and these observations are similar to the results obtained in pigs were such correlations also were not found. However, in the previous study on pigs, serum IGF-1 concentration was associated with the final body weight [18]. Lack of correlations of MvBMD and Ca-HA with all the other investigated parameters were stated in this study that is partially in agreement with studies on pigs, where MvBMD of mandible were not correlated with final body weight, bone weight, bone length and Bvol. However, except for the insignificant correlation between Bvol and Cd in pigs, all the other evaluated parameters of mandible were significantly positively correlated [10]. The current study revealed also that mandibular weight and volume are positively correlated with morphometric, densitometric and mechanical properties of mandible, while mandibular length, A and Ix were correlated only with morphometric (bone weight and volume) and densitometric (Cd) indices. Volumetric bone mineral density of the cortical bone was positively correlated with morphometric parameters (bone weight, length, Bvol, A and Ix) and has shown the most prognostic value for bone mechanical strength prediction, since its value was positively correlated with both Wy and Wf. Besides listed above relationships, significant positive correlations between geometrical (A and Ix as well as MRWT and CI) and mechanical (Wy and Wf) parameters of mandible were stated.

The results obtained in this study indicate that mandible in sheep provide possibility to investigate effects of experimental manipulations on bone tissue metabolism and skeletal system properties with a use of physiological, nutritional, pharmacological, toxicological and environmental factors. Even though mandibular samples were harvested from ram lambs in this study, the same analytical procedures may be applied for mandible from ewes. Except for ovariectomy-induced osteopenia and osteoporosis, experimental evaluation of mandible from ram lambs may provide valuable data on prevention and treatment of metabolic bone disorders resulting from malnutrition, glucocorticoid treatment, androgen deficiency, mineral and vitamin deficiency and impaired environmental conditions. Metabolic effects of positive or negative factors influencing bone tissue properties in mammals may be more apparent when acting during systemic growth and development period than later in skeletally matured or older animals [25]. The elaborated experimental model in this study is characterized by numerous advantages in comparison to other animal models. In rodents, serial blood collection, small size of mandible and existing differences in bone growth and mineralization pattern in comparison to humans make these experimental models and data interpretation less attractive than in case of sheep or other small ruminants [26–29]. Bone size, histological characteristic and bone remodeling activity, as well as bone metabolism responses to hormonal manipulations in sheep skeleton is similar to humans [30, 31]. Moreover, in sheep serial blood sampling, housing, handling and experimental procedures are relatively easy to perform and similarity of the hormonal profile with humans favors sheep model for studies on bone metabolism [32, 33]. The other rationale for the use of mandible in experimental studies on bone metabolism regulation results from impact of bone loss in maxillofacial bones on health and quality of the stomatognathic system and whole body both in animals and humans. Maxillar and mandibular bone loss most commonly results from periodontal disease, resorption of alveolar ridges following tooth extraction, or a combination of both [34]. Apart from these similarities, the differences such as gastrointestinal structure and digestion process in ruminants and quadruped posture must be also considered as potential limitations in some experimental approaches using the sheep model. However, during the neonatal and postnatal period of systemic growth and development, gastrointestinal tract anatomy and digestion and absorption processes in sheep are similar to monogastric mammals like pigs, dogs and humans. The quadruped posture in sheep appears not to be a limitation in studies on bone metabolism regulation and bone quality with the use of mandibular model, since the body posture has no impact on craniofacial bones characteristics [13, 23, 33].

## Conclusions

In conclusion, this study presents basic morphological, densitometric and mechanical characteristics of mandible in 5-month-old Polish Merino sheep. Significant differences of the evaluated parameters between the right and left halves of the mandible were not shown. Common relationships between morphological, densitometric and mechanical parameters of the mandible indicate that the originally elaborated experimental model may serve for further studies on metabolic responses of bone tissue and skeletal system to physiological, nutritional, pharmacological, toxicological and environmental factors influencing bone tissue metabolism. Moreover, quantitative computed tomography may be also used for in vivo and ex vivo evaluation of morphometric and densitometric properties of bones of the stomatognathic system in sheep.

## Abbreviations

A: Cross-sectional area
BAP: Bone-specific alkaline phosphatase
Bvol: Total bone volume
Ca-HA: Calcium hydroxyapatite
Cb_Ca-HA_: Calcium hydroxyapatite density of the cortical bone
Cd: Volumetric bone mineral density of the cortical bone
CI: Cortical index
IGF-I: Insulin-like growth factor I
Ix: Second moment of inertia
MRWT: Mean relative wall thickness
MvBMD: Mean volumetric bone mineral density
PBM: Peak bone mass
QCT: Quantitative computed tomography
vBMD: Volumetric bone mineral density
VOI: Volume-of-interest
Wf: Ultimate strength
Wy: Maximum elastic strength
